# Genomic Risk Factors Driving Immune-Mediated Delayed Drug Hypersensitivity Reactions

**DOI:** 10.3389/fgene.2021.641905

**Published:** 2021-04-16

**Authors:** Yueran Li, Pooja Deshpande, Rebecca J. Hertzman, Amy M. Palubinsky, Andrew Gibson, Elizabeth J. Phillips

**Affiliations:** ^1^Institute for Immunology and Infectious Diseases, Murdoch University, Murdoch, WA, Australia; ^2^Department of Medicine, Vanderbilt University Medical Centre, Nashville, TN, United States

**Keywords:** delayed hypersensitivity, human leukocyte antigen, T-cell receptor, endoplasmic reticulum aminopeptidase, genetic risk, immune checkpoint

## Abstract

Adverse drug reactions (ADRs) remain associated with significant mortality. Delayed hypersensitivity reactions (DHRs) that occur greater than 6 h following drug administration are T-cell mediated with many severe DHRs now associated with human leukocyte antigen (HLA) risk alleles, opening pathways for clinical prediction and prevention. However, incomplete negative predictive value (NPV), low positive predictive value (PPV), and a large number needed to test (NNT) to prevent one case have practically prevented large-scale and cost-effective screening implementation. Additional factors outside of HLA contributing to risk of severe T-cell-mediated DHRs include variation in drug metabolism, T-cell receptor (TCR) specificity, and, most recently, HLA-presented immunopeptidome-processing efficiencies via endoplasmic reticulum aminopeptidase (ERAP). Active research continues toward identification of other highly polymorphic factors likely to impose risk. These include those previously associated with T-cell-mediated HLA-associated infectious or auto-immune disease such as Killer cell immunoglobulin-like receptors (KIR), epistatically linked with HLA class I to regulate NK- and T-cell-mediated cytotoxic degranulation, and co-inhibitory signaling pathways for which therapeutic blockade in cancer immunotherapy is now associated with an increased incidence of DHRs. As such, the field now recognizes that susceptibility is not simply a static product of genetics but that individuals may experience dynamic risk, skewed toward immune activation through therapeutic interventions and epigenetic modifications driven by ecological exposures. This review provides an updated overview of current and proposed genetic factors thought to predispose risk for severe T-cell-mediated DHRs.

## Introduction

**FIGURE 1:**
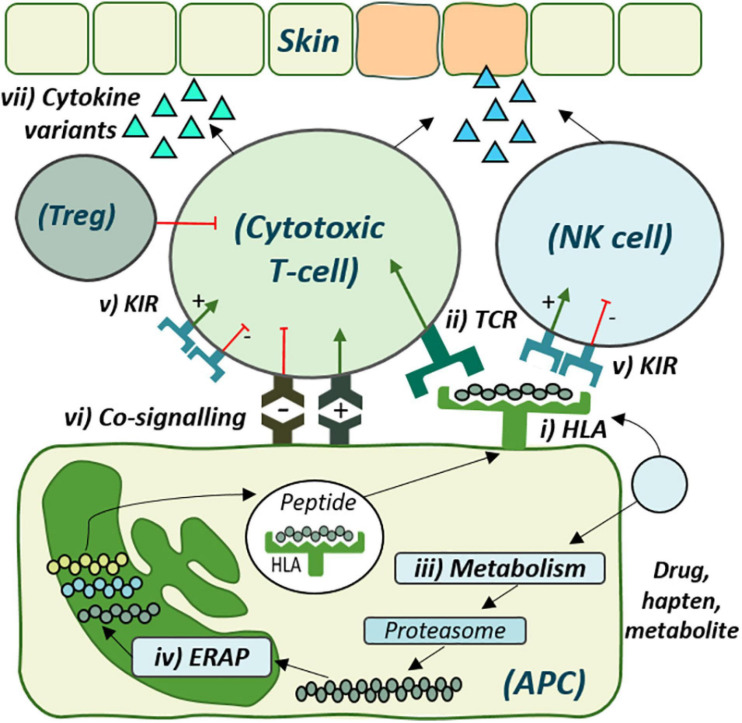
The cellular role of genetic risk factors defined and proposed for development of delayed drug hypersensitivity reactions. Drug antigen may bind directly to (i) specific human leukocyte antigen (HLA) molecules on the surface of antigen-presenting cells (APC) for recognition by corresponding (ii) T-cell receptors (TCRs). Alternatively, drug may undergo (iii) metabolism and cellular processing by (iv) endoplasmic reticulum aminopeptidase (ERAP), with the peptide products then presented by the risk HLA molecule for TCR recognition. (v) Distinct killer-cell immunoglobulin-like receptors (KIR), expressed on both T-cell and Natural Killer (NK) cells, also show specificity for HLA-antigen interaction, which may regulate cytotoxic degranulation. (vi) Co-signaling pathways also regulate T-cell activation, with overall co-inhibition or co-stimulation leading to (vii) cytokine release and respective tolerance with T-regulatory cell (Treg)-induced immunosuppression or inflammation and cytotoxic degranulation, respectively.

Adverse drug reactions (ADRs) are estimated as the fourth to sixth leading cause of death (Dormann et al., 2000; Pouyanne et al., 2000; Miya et al., 2019). While the majority are classified as type A, predictable based on drug pharmacology, the remainder are off-target type B ADRs and inclusive of T-cell-mediated delayed drug hypersensitivity reactions (DHRs). While DHRs may elicit systemic effects, diverse clinical reactions also target specific organs including drug-induced liver injury (DILI), associated with nausea, fatigue, jaundice, and mortality up to 9.4% (Leise et al., 2014). However, most often they target skin, with presentation from mild rash (fixed drug eruption, maculopapular exanthema) to life-threatening severe cutaneous adverse reactions (SCARs) including Stevens-Johnson Syndrome/Toxic Epidermal Necrolysis (SJS/TEN) and drug reaction with eosinophilia and systemic symptoms (DRESS) (Peter et al., 2017). DRESS has a mortality up to 10% (Kardaun, 2019; Wolfson et al., 2019) and is characterized by widespread skin eruption, lymphadenopathy, fever, and multiple organ involvement (Choudhary et al., 2013; Kardaun, 2019). SJS and TEN are the same disease across a spectrum of severity with the higher end of mortality (TEN) associated with up to 50% death (Patel et al., 2013; Langley et al., 2018). SJS/TEN is characterized by blistering and involvement of at least two mucous membranes (Paulmann and Mockenhaupt, 2015; Miya et al., 2019; Zimmerman and Dang, 2020). Despite clinical distinction, lack of mechanistic delineation has precluded development of disease-specific treatment and prevention strategies (Pavlos et al., 2015; Redwood et al., 2018). In recent years many DHRs have been associated with strong human leukocyte antigen (HLA) class I associations opening pathways to prediction and prevention (Figure 1).

## The Evolving Complexity of Drug-, Reaction-, and Population-Restricted HLA Risk

### Abacavir Hypersensitivity

The HLA locus is highly polymorphic with >25,000 allelic variants annotated (HLA.alleles.org). In 2002, Mallal demonstrated carriage of HLA-B^∗^57:01 among 78% of HIV patients with abacavir hypersensitivity, which is a well-characterized systemic syndrome, opposed to just 2% of tolerant patients (Mallal et al., 2002). A randomized double blind clinical trial of real-time HLA-B^∗^57:01 screening versus abacavir treatment without real-time screening showed a negative predictive value (NPV) of 100% and a positive predictive value (PPV) of 55% (Mallal et al., 2008), demonstrating that HLA-B^∗^57:01 screening eliminates patch test positive abacavir hypersensitivity. This PREDICT-1 study was the licensing study upon which guideline-based HLA-B^∗^57:01 screening prior to abacavir prescription was established.

### Carbamazepine Hypersensitivity

In 2004, association between HLA-B^∗^15:02 and carbamazepine (CBZ)-induced SJS/TEN in Taiwan was reported, which followed the translational roadmap provided by abacavir such that 0/4120 Taiwanese HLA-B^∗^15:02-negative patients developed SJS/TEN after CBZ exposure (Chung et al., 2004). Pre-prescription HLA-B^∗^15:02 screening for CBZ is now active in Hong Kong, Singapore, and Thailand where there is high allelic prevalence (FerrellJr., and McLeod, 2008). However, HLA-B^∗^15:02 is expressed in <1% of patients of European or African ancestry despite global disease burden, restricting universal screening and inferring that different HLA alleles drive reactions in different populations (Karnes et al., 2019). Indeed, multiple alleles are now associated with CBZ-SCAR in distinct populations, with HLA-A^∗^31:01 associated with DRESS in Europeans and Chinese, but not SJS/TEN (McCormack et al., 2011; Genin et al., 2014), highlighting propensity for distinct alleles to define risk for specific reactions. Most recently, Nicoletti reported HLA-A^∗^31:01 as a strong risk factor broadly across CBZ-induced SCAR and DILI in Europeans (Nicoletti et al., 2019) while Mockenhaupt described an HLA-B^∗^57:01 association for CBZ-SJS/TEN in Europeans (Mockenhaupt et al., 2019). These studies demonstrate that HLA restriction may be complex, with influence from multiple alleles restricted to antigen, reaction phenotype, and population (Table 1).

**TABLE 1:** HLA risk alleles associated with delayed type drug hypersensitivity reactions.

Drug	HLA risk allele	Reaction	Ethnic population	PPV (NPV)	References
Abacavir	B*57:01	HSS	African	50 (100)	Saag et al., 2008
			Caucasian	50 (100)	Mallal et al., 2002, 2008
			Hispanic	96 (60)	Sousa-Pinto et al., 2015
Acetazolamide	B*59	SJS/TEN	Korean		Her et al., 2011
Allopurinol	B*58:01	DRESS, SJS/TEN	Caucasian		Jarjour et al., 2015
		DRESS	Caucasian (Portuguese)		Gonçalo et al., 2013
		DRESS, SJS/TEN	Han Chinese	3 (100)	Chiu et al., 2012
		DRESS, SJS/TEN	Korean	2.06 (99.98)	Kang et al., 2011
		DRESS	Thai	8.26 (100)	Sukasem et al., 2016
		MPE, SJS/TEN	Japanese		Kaniwa et al., 2008; Jarjour et al., 2015
		MPE	Thai	5.13 (99.90)	Sukasem et al., 2016
		SJS/TEN	Caucasian		Lonjou et al., 2008; Yu et al., 2017
			Thai	10.48 (100)	Sukasem et al., 2016
	C*03:02	DRESS, SJS/TEN	Korean	1.77 (99.98)	Kang et al., 2011
	A*33:02	DRESS, SJS/TEN	Korean	0.8 (99.96)	Kang et al., 2011
Amoxicillin-clavulanate	DRB1*15:01	DILI	Caucasian		Lucena et al., 2011
Azathioprine	DQA1*02:01	Pancreatitis	Caucasian		Heap et al., 2014
	DRB1*07:01				Heap et al., 2014
Benznidazole	A*11:01	MPE, DRESS	Bolivian	100 (70)	Balas et al., 2020
	A*29:02			100 (70)	
	A*68			48 (84)	
Carbamazepine	A*24:02	SJS/TEN	Han Chinese		Shi et al., 2012
	A*31	DRESS, SJS/TEN, MPE	Japanese		Niihara et al., 2012
	A*31:01	DRESS	Caucasian	0.77 (99.98)	Genin et al., 2014
			Han Chinese	0.67 (99.97)	Genin et al., 2014
		SJS/TEN	Caucasian		McCormack et al., 2011
			Han Chinese		Genin et al., 2014
		DRESS, SJS/TEN	Korean		Kim et al., 2011b
		SCAR, DILI	Caucasian		Nicoletti et al., 2019)
	B*15:02	SJS/TEN	Han Chinese	2.24 (99.94)	Tangamornsuksan et al., 2013; Genin et al., 2014
			Indian		Mehta et al., 2009
			Korean		Tangamornsuksan et al., 2013
			Malaysian		Tangamornsuksan et al., 2013
			Thai		Tangamornsuksan et al., 2013; Sukasem et al., 2018
			Taiwanese	93.6 (100)	Chung et al., 2004
	B*15:11	SJS/TEN	Han Chinese		Shi et al., 2012
			Asian	43.8 (95.1)	Wang et al., 2017
	B*15:21	SJS/TEN	Thai		Sukasem et al., 2018
		SJS/TEN	Filipino	1.03 (87.5)	Capule et al., 2020
	B*51:01	DRESS, MPE	Han Chinese		Wang et al., 2017
	B*57:01	SJS/TEN	Caucasian		Mockenhaupt et al., 2019
	B*58:01	DRESS, MPE	Asian	90.4 (37)	Wang et al., 2017; Sukasem et al., 2018
	DRB1*14:05	MPE	Han Chinese		Li et al., 2013
Co-trimoxazole	B*15:02, C*08:01	SJS/TEN	Thai		Sukasem et al., 2020
	B*13:01	DRESS			
Dapsone	B*13:01	DRESS	Chinese	7.8 (99.8)	Zhang et al., 2013
		DRESS, SJS/TEN	Thai		Tempark et al., 2017
		DRESS	Taiwanese		Chen et al., 2018
			Malaysian		
Flucloxacillin	B*57:01	DILI	Caucasian	0.12 (99.99)	Daly et al., 2009
Isoxicam, Piroxicam	A*02	SJS/TEN	Caucasian		Roujeau et al., 1987
	B*12				
Lamotrigine	A*02:07	MPE, DRESS, SJS/TEN	Thai		Koomdee et al., 2017
	A*24:02, C*01:02	MPE	Korean		Moon et al., 2015
	A*30:01		Han Chinese		Li et al., 2013
	B*13:02				
	A*33:03		Thai		Koomdee et al., 2017
	B*44:03				
	A*31:01	DRESS, SJS/TEN	Korean		Kim et al., 2017
	A*68:01	DRESS, SJS/TEN	Caucasian		Kazeem et al., 2009
	B*15:02	SJS/TEN	Han Chinese		Cheung et al., 2013
		DRESS, SJS/TEN, MPE	Thai		Koomdee et al., 2017
		SJS/TEN	Iranian	78.57 (56.41)	Sabourirad et al., 2020
	B*38	SJS/TEN	Caucasian		Lonjou et al., 2008
	B*58:01	DRESS, SJS/TEN	Caucasian		Kazeem et al., 2009
	C*07:18				
	DQB1*06				
	DRB1*13				
Methazolamide	B*59:01	SJS/TEN	Japanese		Nakatani et al., 2019
			Korean		Tangamornsuksan and Lohitnavy, 2019
			Han Chinese	100 (96.8)	Yang et al., 2015; Tangamornsuksan and Lohitnavy, 2019
Minocycline	B*35:02	DILI	Caucasian		(Urban et al., 2017)
Nevirapine	Cw4	DRESS	Han Chinese		Gao et al., 2012
	C*04:01	SJS/TEN	Malawian	2.6 (99.2)	Carr et al., 2013, 2017
	C*08	DRESS	Japanese		Gatanaga et al., 2007
	C*08:02, B*14:02	DRESS	Caucasian (Sardinian)		Littera et al., 2006
	B*35:05	Skin Rash	Thai		Chantarangsu et al., 2009
	DRB1*01:01	DRESS	Caucasian		Martin et al., 2005
Oxcarbazepine	A*03:01	MPE	Uighur Chinese		Zhao et al., 2020
	B*07:02				
	B*15:02	MPE, SJS/TEN	Han Chinese		Hung et al., 2010
	B*38:02	MPE			Lv et al., 2013
Oxicams	B*73	SJS/TEN	Caucasian		Lonjou et al., 2008
Phenobarbital	B*51:01	SJS/TEN	Japanese		Kaniwa et al., 2013
Phenytoin	B*13:01	SJS/TEN	East Asian		Su et al., 2019
	B*15:02	SJS/TEN	East Asian		Su et al., 2019
			Han Chinese		(Cheung et al., 2013
			Malaysian		(Chang et al., 2017
			Thai	33 (100)	Locharernkul et al., 2008
	B*15:13	DRESS, SJS/TEN	Malaysian		Chang et al., 2017
	B*56:02	SJS/TEN	Thai		Tassaneeyakul et al., 2016
		DRESS	Australian Aboriginal		Somogyi et al., 2019
	Cw*08:01	SJS/TEN	Han Chinese		Hung et al., 2010
	DRB1*16:02				
Raltegravir	B*53:01	DRESS	African		Thomas et al., 2017
Strontium Renalate	A*33:03	SJS/TEN	Han Chinese		Lee et al., 2016
	B*58:01				
Sulfamethoxazole	A*29	SJS/TEN	Caucasian		Roujeau et al., 1987
	A*30	FDE	Turkish		Özkaya-Bayazit and Akar, 2001
	A*30-B*13-C*06				
	A*11:01	SJS/DRESS	Japanese		Nakamura et al., 2020
	B*13:01	SCAR	Asian	4.05 (99.92)	Wang et al., 2020
		DRESS		3.64 (99.92)	
	B*14:01	DILI	European American		Li et al., 2020
	B*35:01		African American		Li et al., 2020
	B*44 (B12 serotype)	SJS/TEN	Caucasian		Liang et al., 2013
	B*38	SJS/TEN	Caucasian		Lonjou et al., 2008
	DR*07				
Sulfasalazine	B*13:01	DRESS	Han Chinese		Yang et al., 2014
Ticlopidine	A*33:03	DILI	Japanese		Hirata et al., 2008
Terbinafine	A*33:01	DILI	Caucasian		Fontana et al., 2018
Vancomycin	A*32:01	DRESS	Caucasian		Konvinse et al., 2019
Zonisamide	A*02:07	SJS/TEN	Japanese		Kaniwa et al., 2013

*References included were studies associated with clinically defined DHR. DILI, drug-induced liver injury; DRESS, drug reaction with eosinophilia and systemic symptoms; FDE, fixed drug eruption; HSS, hypersensitivity syndrome; MPE, maculopapular eruption; NPV, negative predictive value; PPV, positive predictive value; SCAR, severe cutaneous adverse reaction; SJS/TEN, Stevens-Johnson Syndrome/Toxic Epidermal Necrolysis. NPV and PPV are based on case-control studies and require ongoing validation and thus subject to change.*

## HLA and Its Use in Clinical Practice

### HLA-B^∗^58:01 and Allopurinol-DRESS

Other strong HLA associations have been described with near-complete NPV for WHO essential medicines, the most effective and safe drugs to meet the most important needs, such as allopurinol, dapsone, and vancomycin (WHO, 2021). Allopurinol is used for treatment of gout but is also the most prevalent drug cause of DRESS in the FDA Adverse event reporting system (Bluestein et al., 2021). In 2005, HLA-B^∗^58:01 was associated with allopurinol-induced SCAR with 100% NPV in Southeast Asians (Hung et al., 2005). Subsequent studies confirmed risk in cohorts from Europe (Lonjou et al., 2008), Japan (Kaniwa et al., 2008), Thailand (Yu et al., 2017), South Korea (Kang et al., 2011), and Portugal (Gonçalo et al., 2013), but, as with CBZ, comparative strength of association and allelic frequency is not replicated and is far lower in Europeans (Génin et al., 2011). Currently, where patients are known to be HLA-B^∗^58:01+, the European Medicines Agency advises clinicians to avoid allopurinol and screening is recommended in Korean, Thai, or Han Chinese patients (Ke et al., 2017). However, recent analysis in the UK defined the number needed to test (NNT) as 11,286, leading the panel to advise against routine screening (Plumpton et al., 2017).

### HLA-B^∗^13:01 and Dapsone-SCAR

The antibiotic dapsone is predominantly associated with treatment of leprosy (Wolf et al., 2002). In 2013, HLA-B^∗^13:01 was described with 99.8% NPV and 7.8% PPV as a risk factor among Chinese patients for dapsone hypersensitivity (Zhang et al., 2013). While prevalent in Chinese and Indian populations, HLA-B^∗^13:01 is comparatively absent among Europeans and Africans. HLA-B^∗^13:01 risk is now confirmed for dapsone-SCAR in Thailand (Tempark et al., 2017) and research has modeled drug interaction within the HLA binding site (Watanabe et al., 2017). Most recently, Chen expanded HLA-B^∗^13:01 risk to patients from Malaysia and Taiwan (Chen et al., 2018), and Zhao identified dapsone-responsive HLA-B^∗^13:01-restricted CD8^+^ T-cells in patients (Zhao et al., 2019).

### HLA-A^∗^32:01 and Vancomycin-DRESS

Vancomycin, a front-line treatment for beta-lactam-resistant infections (Rybak et al., 2009; Frymoyer et al., 2013; Moore et al., 2020), is the most common antibiotic instigator of DRESS (Wolfson et al., 2019). In 2019, Konvinse published strong association between HLA-A^∗^32:01 and vancomycin-DRESS determining that 20% of HLA-A^∗^32:01+ patients would develop the disease (Konvinse et al., 2019). With a European prevalence of 6.8%, they predicted the NNT as just 75 and have since developed an HLA-A^∗^32:01-specific, cost-effective real-time PCR screen (Rwandamuriye et al., 2019). In 2020, Nakkam described cross-reactivity with an alternate glycopeptide antibiotic, teicoplanin, in 16% of HLA-A^∗^32:01+ vancomycin-DRESS patients predicted by a shared class II HLA haplotype (Nakkam et al., 2020). These data implicate risk alleles with influence not simply to dictate predisposition but with ramifications for ongoing treatment. Importantly, while predictive values defined by limited case-control studies may not be indicative of risk in the underlying population, warranting caution, in vitro assays have functionally confirmed that HLA risk restricted drug-specific T-cell activation for abacavir, CBZ, allopurinol, dapsone, and vancomycin (Chessman et al., 2008; Wei et al., 2012; Yun et al., 2014; Zhao et al., 2019; Nakkam et al., 2020).

## Recently Reported HLA Associations (2019-)

Single HLA associations up until 2019 have been extensively reviewed (White et al., 2015; Karnes et al., 2019; Oussalah et al., 2020). Since then, further advancement in sequencing platforms has been providing increased resolution that has enabled discovery of novel HLA associations (LaHaye et al., 2016; van der Ven et al., 2018; Giannopoulou et al., 2019; Mimori et al., 2019). In 2019, Nakatani reported a Japanese association between SJS/TEN, HLA-A^∗^02:06:01, and cold medicines containing non-steroidal anti-inflammatories (Nakatani et al., 2019). Furthermore, Tangamornsuksan reported an association between methazolamide-induced SJS/TEN and HLA-B^∗^59:01 in Koreans and Han Chinese (Tangamornsuksan and Lohitnavy, 2019). In 2020, within a Thai HIV cohort, Sukasem reported an association between co-trimoxazole-induced DRESS with HLA-B^∗^13:01 and SJS/TEN with HLA-B^∗^15:02 and HLA-C^∗^08:01 (Sukasem et al., 2020). Furthermore, MPE and DRESS resulting from benznidazole was associated with HLA-A^∗^68, A^∗^11:01, and A^∗^29:02 in Bolivian patients with Chagas disease (Balas et al., 2020). Most recently, Zhao reported an association between oxcarbazepine-induced MPE and HLA-A^∗^03:01 and HLA-B^∗^07:02 in patients of Uighur Chinese ethnicity (Zhao et al., 2020). Moreover, HLA associations have also been reported for herbal medicines including green tea (Hoofnagle et al., 2020) and polygonum multiflorum with HLA-B^∗^35:01 (Li et al., 2019). These studies provide a glimpse into the recent progress toward risk prediction specific to populations, yet a significant hurdle remains risk discovery in minority groups for whom access to large cohorts for traditional population studies is nearly impossible. One strategy is to maximize utility of international SCAR registries where careful patient matching for drug, reaction phenotype, and ethnicity may provide means to explore shared risk (Somogyi et al., 2019). Indeed, Somogyi identified three patients of Australian Indigenous ethnicity with phenytoin-DRESS sharing HLA-B^∗^56:02 (Somogyi et al., 2019). Critically, HLA-B^∗^56:02 frequency ranges up to 19% in this population but is absent from the predominant Australian European populace, highlighting utility of detailed biobanking with functional validation of proposed risk alleles (Monshi et al., 2013; Pan et al., 2019). Another possibility is the likelihood that alleles with shared specificities drive response to the same drug, as for nevirapine (Chantarangsu et al., 2009; Carr et al., 2013). Here, association with HLA-C^∗^04 across ethnicities is driven by a unique F pocket motif that determines similar binding specificity for HLA-C^∗^04:01 with HLA-C^∗^05:01 and HLA-C^∗^18:01, dominant in Hispanics and Africans, respectively (Pavlos et al., 2017). The ability to design HLA crystal structures combined with HLA binding algorithms provides a functional bridge to understand whether proposed antigen binds to diverse alleles (Pavlos et al., 2017). Nonetheless, HLA is not the sole requirement for T-cell activation and other parameters are proposed to retain the HLA-restricted “positive predictive gap.”

## T-Cell Receptors Provide Specificity for Recognition of Risk HLA-Antigen Complex

Antigenic peptides bound to HLA must contact the T-cell receptor (TCR) to trigger T-cell activation (Figure 1). Each individual’s TCR repertoire comprises a diverse blend of public and private TCRs, which, through prior antigen exposure, may be uniquely distributed in tissues (Robins et al., 2010). A polyclonal response is well documented for abacavir (Redwood et al., 2019). This is in keeping with the altered peptide repertoire hypothesis suggesting that abacavir binds within the F pocket of the HLA-B^∗^57:01 peptide binding groove altering its peptide specificity and the repertoire of self-peptides recognized as immunogenic (Illing et al., 2012). Polyclonal response is also observed during CDR3 spectratyping after the *in vitro* priming of naïve T-cells to the immunogenic drug metabolite sulfamethoxazole-nitroso (SMX-NO) (Gibson et al., 2017). Here the authors implicate the high protein reactivity of SMX-NO thought to drive formation of multiple haptens, each with potential to produce a diverse array of antigenic peptides. However, early work by Nassif reports predominant expression of Vβ 13.1 and 14 on T-cells in the blister of such patients, suggesting that early response in tissue is driven by more select, dominant clonotypes (Nassif et al., 2002). In 2019, Pan reported dominant single, public “VFDNTDKLI” TCRα CDR3 and “ASSLAGELFF” TCRβ CDR3 in HLA-B^∗^15:02+ patients with CBZ hypersensitivity, rare in blood but dominantly expressed in blister (Pan et al., 2019). The dominant TCR was identified on T-cells expressing granulysin, a key cytotoxic mediator with precedent in eliciting tissue damage (Pan et al., 2019). Furthermore, the complete TCR blueprint provided by single-cell sequencing was synthetically reconstructed and shown to trigger T-cell activation specific to CBZ and HLA-B^∗^15:02. Preferential TCR expansion has also been described in blister during HLA-B^∗^58:01-associated allopurinol-SCAR (Chung et al., 2015). While further studies are warranted, those described begin to elucidate the specificity of a single dominantly expanded TCR to drive early response in the tissue of HLA-predisposed patients.

## Erap Variants Skew the HLA-Restricted Immunopeptidome

Although drug-protein conjugates are found at similar levels in allergic and tolerant patients (Park et al., 1998; Sullivan et al., 2015), the downstream impact of N-terminal peptide trimming that shapes the HLA-presented immunopeptidome has remained undefined. This process is performed by endoplasmic reticulum aminopeptidases (ERAPs) 1 and 2 (Serwold et al., 2001; Chang et al., 2005; Figure 1) for which polymorphic variants alter susceptibility and outcome to autoimmune disease and viral infections with HLA class I-restricted etiologies (Evans et al., 2011; Guerini et al., 2012; Biasin et al., 2013; Fruci et al., 2014; Reeves and James, 2015; Saulle et al., 2019; Vidal-Castiñeira et al., 2020). Specifically, distinct ERAP1 allotypes skew the HLA-class I-expressed immunopeptidome during infectious disease, where hypoactive allotypes result in longer sub-dominant peptides that impair CD8^+^ T-cell response (Kemming et al., 2019). Intriguingly, peptides with aromatic or hydrophobic C-terminal amino acids are favored by ERAP1 for efficient N-terminal trimming and treatment of cells with abacavir alters the self-peptide preference toward the same amino acids (Chang et al., 2005; Ostrov et al., 2012). In 2020, Pavlos identified ERAP1 as a novel predictor of abacavir tolerance among HLA-B^∗^57:01+ patients. Tolerant patients were significantly more likely to express ERAP1 hypoactive allotypes with reduced trimming efficiency compared to hypersensitive patients (Pavlos et al., 2020). While yet to transverse other drugs, the epistatic relationship between HLA and ERAP raises intrigue to the influence of other such genes. One such entity is the highly polymorphic Killer-cell Immunoglobulin-like receptors (KIRs) expressed on T-cells and Natural Killer (NK) cells (Mingari et al., 1997; LeMaoult et al., 2005), with both cell types reporting the predominant infiltrate of in SJS/TEN blister (Chung and Hung, 2010). HLA alleles are the distinct ligands for KIRs that regulate cytotoxic degranulation in a complex interaction with sensitivity to the presented peptide via overlapped TCR binding (Mandelboim et al., 1997; Boyington and Sun, 2002; Thananchai et al., 2007; Fadda et al., 2010; Figure 1). Notably, specific KIR have been associated with progression of HLA-restricted infectious disease (Bellón, 2019). Description by Fasbender of the induction of NK-activating ligands on hepatocytes after drug exposure, driving NK-mediated cytotoxicity, spurs interest given that T-cells in the blood of SJS/TEN patients overexpress KIR2DL2 and KIR2DL3 (Morel et al., 2010; Fasbender et al., 2020). With yet unreported genetic or functional assessment, studies are warranted to understand the combined influence of these interactions.

## The Limited Role of Altered Drug Metabolism in Formation of Immunogenic Moieties

Drugs lacking protein reactivity may directly activate T-cells (Schnyder et al., 1997; Zanni et al., 1997; Naisbitt et al., 2003). However, metabolic detoxification pathways form protein-reactive metabolites, also reported to activate drug-specific T-cells (Naisbitt et al., 2001; Sullivan et al., 2015; Figure 1). Metabolism is highly varied due to polymorphic enzymes, with cytochrome P450 (CYP450) enzymes responsible for 90% of drug metabolism (Lynch and Price, 2007) and for which allelic variants are described from poor to ultrarapid metabolisers (Zanger and Schwab, 2013). While metabolic activity of skin is considered limited (Sharma et al., 2019), keratinocytes show capacity to metabolize and present drug-derived antigens (Reilly et al., 2000; Roychowdhury and Svensson, 2005). Several studies now investigate metabolic variants associated with DHR, most notably for phenytoin, predominantly oxidized to an inactive metabolite by CYP2C9 with minor contribution by CYP2C19. Genetic analyses show that CYP2C9^∗^2 and CYP2C9^∗^3 low function variants extend exposure to the immunogenic parent drug (Aynacioglu et al., 1999; Silvado et al., 2018). Specifically, CYP2C9^∗^3 is associated with SJS/TEN in both Han Chinese (Chung et al., 2014) and Thai (Suvichapanich et al., 2015; Tassaneeyakul et al., 2016). In addition, CYP2C19^∗^3 is associated with phenytoin-DRESS in Thai (Yampayon et al., 2017). In 2019, Su et al. (2019) published on the utility of combined risk HLA and CYP2C9^∗^3 genetic testing in Asian populations to prevent phenytoin hypersensitivity. It is now advised that physicians reduce the starting dose by 25% for patients classed as intermediate metabolizers, defined by CYP2C9^∗^1/^∗^3 and CYP2C9^∗^1/^∗^2 carriage (Caudle et al., 2014). Metabolic variation is also associated with DHR driven by nevirapine, hydroxylated by CYP2B6. Loss of functional alleles CYP2B6^∗^6 and CYP2B6^∗^18 are associated with increased susceptibility for nevirapine-SJS/TEN, with the ^∗^18 variant only observed in patients of African ancestry (Ciccacci et al., 2013; Carr et al., 2014). A handful of other associations are explored by Pirmohamed and were not significant upon multiple-testing correction (Pirmohamed et al., 2000); thus, most data to date implicate only a minor role for metabolic variation in DHR.

## The Influence of Infectious Disease

There are three main aspects to consider for the impact of infectious disease on DHR. The first aspect is the effect of cumulative drug exposure in cohorts where long-term exposures are driven by repeat infection like antibiotic hypersensitivity in patients with cystic fibrosis (CF). Indeed, CF patients are far more likely to develop an allergy to beta-lactams than patients without (Burrows et al., 2007; Wright et al., 2018); thus, it is possible that repeat high dosing and antigen accumulation contributes to risk. Second is the potential for disease-associated immune dysregulation to heighten allergic susceptibility. An example is the reduced DHR incidence in HIV patients following initiation of successful highly active antiretroviral therapy, which controls viral progression, preventing deterioration of immune function (Coopman et al., 1993; Li et al., 1998). Similarly, studies show that CF patients have dysfunctional antiviral T-cell responses (Hubeau et al., 2004). Indeed, toll-like receptor 4, which mediates inflammatory cytokine expression, is reduced in CF airway cell lines (John et al., 2010; Keiser et al., 2015). Interestingly, cytokine variants predispose to DHRs such as liver injury: IL10-592 AA and IL10-819 TT are associated with docetaxel-induced liver injury, and polymorphism-380G/A in TNF−α is associated with hepatitis induced by antituberculosis drugs (Kim et al., 2011a; Liang et al., 2013; Figure 1). Evidence suggests that drug antigens may mount response in tissue through pre-existing antiviral T-cells in a heterologous immunity model (Descamps et al., 2003; Mitani et al., 2005). Functional evidence is based on work by Lucas who showed that all drug-naive HLA-B^∗^57:01+ individuals have T-cells responsive to abacavir (Lucas et al., 2015; Gibson et al., 2017). Such reactive promiscuity across all healthy donors implicates cross-reactivity with common broad-exposure pathogens (Smith et al., 2016).

## The Inferred Role of Epigenetic Risk

It is now well established that epigenetic modifications to open or close the transcriptional template of genes impacts immunological processes (North and Ellis, 2011; Moggs et al., 2012). Epigenetic influence is environmental with documented effects from diet, viral exposures, and pollution driving distinguishable differences in immune status; thus, it may drive not only inter-individual but also intra-individual risk over time, proposing dynamic susceptibility. Indeed, Nadeau describes hypermethylation of the FOXP3 locus affecting Treg function and asthma severity in patients who live in areas with higher air pollution (Nadeau et al., 2010). Evidence now suggests that epigenetic effects may be multi-generational, with lead exposure and subsequent DNA methylation of fetal germ cells in grandparents traced through to grandchildren (Sen et al., 2015). While likely, epigenetic influence has yet to be directly inferred upon susceptibility to DHR, but there is some initial evidence. In 2018, Cheng published that risk of allopurinol-induced SCAR was attributed to variants of HCP5, PSORS1C1, TSHZ2, and NOTCH4. Although distinct polymorphisms and thus genetic variants, intriguingly NOTCH4 and TSHZ2, were included as genes that presented as highly differentially methylated, a form of epigenetic regulation (Cheng et al., 2017). Furthermore, Monroy-Arreola demonstrated upregulation of microRNA-21, -18, and -155 in drug-specific CD4+ T-cells from hypersensitive patients (Monroy-Arreola et al., 2018). While microRNA may regulate post-transcriptional gene expression, others bind to control regulators of epigenetic modification including DNA methyltransferases (Sato et al., 2011).

## Dynamic Dysregulation Imposed by Immune Checkpoints Spans Genetic and Therapeutic Risk

Immune checkpoints regulate T-cell activation to prevent uncontrolled activation. This complex process is the summation of varied co-stimulatory and opposingly co-inhibitory pathways (Figure 1). Intriguingly, polymorphic variants of checkpoints are linked to numerous autoimmune diseases including rheumatoid arthritis (Kong et al., 2005), multiple sclerosis (Kroner et al., 2005), and ankylosing spondylitis (Kantarci et al., 2003). While allelic influence is yet to be translated to risk for DHR, mechanistic studies have demonstrated the impact of blocking programmed death-1 (PD-1) or cytotoxic lymphocyte antigen-4 (CTLA4) axes to enhance naive T-cell priming to drug antigens (Gibson et al., 2014, 2017). Checkpoint inhibition is now widely adopted in cancer immunotherapy to re-invigorate anti-tumor T-cell responses, but dysregulation is not antigen-specific and immune-mediated ADR are common (Naidoo et al., 2015; Saw et al., 2017; Lomax et al., 2019). While reactions are varied and typically reported as enhanced immunogenicity to self (Mangan et al., 2020), emerging small cohort studies describe a high incidence of DHR in immune checkpoint inhibitor-treated patients (Imafuku et al., 2017; Ford et al., 2018). These studies remain only clinical observations and distinct checkpoint alleles have not been identified in genome-wide association studies; however, given the influence of multiple, counteracting co-signaling pathways, it may be that single variants have a low individual effect for which the previous studies have been underpowered. Further study is now warranted to define association with a greater risk of drug hypersensitivity reactions.

## Summary

Given a lack of a single HLA allele to provide complete PPV, other risk factors must further restrict response and recent advances have detailed (i) application of single-cell sequencing to define the HLA-restricted dominant TCR driving early response in tissue and (ii) the impact of ERAP variants to skew immunodominant peptide presentation. Intriguingly, other proposed risk factors such as checkpoint receptors span genetic and epigenetic risk, with expression subject to environmental or therapeutic pressures, implicating highly dynamic risk. Strategies are now needed to identify risk alleles in minority populations where large clinical cohorts are impossible to obtain. The availability of multi-omic approaches offers opportunity to merge high-resolution genotyping with single-cell phenotyping to tease out more complex risk signatures that may also enable cost-effective patient screening.

## Author Contributions

YL, PD, RH, and AP contributed writing toward individual sections of the manuscript, led and majority authored by YL. AG and EP provided expert review, direction, and guidance. All authors contributed to the article and approved the submitted version.

## Conflict of Interest

The reviewer AC declared a past co-authorship with the authors AG and EP to the handling editor. EP was Drug Allergy Section Editor and receives royalties from Uptodate and consulting fees from Biocryst, Janssen and Vertex. She is co-director of IIID Pty Ltd that holds a patent for HLA-B*57:01 testing for abacavir hypersensitivity, and she holds a patent for Detection of Human Leukocyte Antigen-A*32:01 in connection with Diagnosing Drug Reaction with Eosinophilia and Systemic Symptoms without any financial remuneration and not directly related to the submitted work. Funders played no role in any aspect of this Review. The remaining authors declare that the research was conducted in the absence of any commercial or financial relationships that could be construed as a potential conflict of interest.
